# Working Memory, Cognitive Load and Cardiorespiratory Fitness: Testing the CRUNCH Model with Near-Infrared Spectroscopy

**DOI:** 10.3390/brainsci9020038

**Published:** 2019-02-09

**Authors:** Nounagnon Frutueux Agbangla, Michel Audiffren, Jean Pylouster, Cédric T. Albinet

**Affiliations:** 1 Centre de Recherches sur la Cognition et l’Apprentissage (UMR 7295), Université de Poitiers and Université François-Rabelais de Tours, 86073 Poitiers CEDEX 9 France; michel.audiffren@univ-poitiers.fr (M.A.); jean.pylouster@univ-poitiers.fr (J.P.); 2 Atelier SHERPAS—Unité de Recherche Pluridisciplinaire Sport Santé Société (EA 7369), Université d’Artois, 62800 Liévin, France; 3 Laboratoire Sciences de la Cognition, Technologie, Ergonomie (SCoTE—EA 7420), Université de Toulouse, INU Champollion, 81012 Albi, France

**Keywords:** fNIRS, aging, cognitive load, cardiorespiratory fitness, cerebral oxygenation, updating of working memory

## Abstract

The present study aimed to examine the effects of chronological age and cardiorespiratory fitness (CRF) on cognitive performance and prefrontal cortex activity, and to test the compensation-related utilization of neural circuits hypothesis (CRUNCH). A total of 19 young adults (18–22 years) and 37 older ones (60–77 years) with a high or low CRF level were recruited to perform a working memory updating task under three different cognitive load conditions. Prefrontal cortex hemodynamic responses were continuously recorded using functional near-infrared spectroscopy, and behavioral performances and perceived difficulty were measured. Results showed that chronological age had deleterious effects on both cognitive performance and prefrontal cortex activation under a higher cognitive load. In older adults, however, higher levels of CRF were related to increased bilateral prefrontal cortex activation patterns that allowed them to sustain better cognitive performances, especially under the highest cognitive load. These results are discussed in the light of the neurocognitive CRUNCH model.

## 1. Introduction

The normal aging process is accompanied by cognitive decline in several domains, especially memory and executive functioning [1,2,3,4]. Numerous neuroimaging studies have suggested that these adverse effects are related to changes in brain activity, often in the direction of an age-related decrease in neural activity during the performance of cognitive tasks [5,6]. By contrast, several studies among older adults have documented either increased activation in some brain areas or else the recruitment of additional brain regions, especially in the frontal lobes [7,8,9]. A particularly consistent finding is that brain activation patterns during the performance of cognitive tasks tend to be less lateralized. This pattern of hemispheric asymmetry reduction in older adults (HAROLD [10]) has been observed in a number of tasks involving memory or executive functions [11,12,13,14,15,16,17,18]. Its functional interpretation, reflecting either dedifferentiation [19] or compensation [17,20,21] is still under debate. To be considered as a form of functional compensation, this over recruitment of contralateral regions would have to be accompanied by and related to preserved or increased cognitive performance (for a recent position paper, see [22]). This was theorized by Reuter-Lorenz and colleagues [23,24], who put forward the compensation-related utilization of neural circuits hypothesis (CRUNCH). According to this neurophysiological model, neural activity varies with the level of task demands or cognitive load. When the cognitive load is low, it predicts overactivation or bilateral recruitment in older adults, mainly in the prefrontal cortex (PFC), contrasting with more focal activation in young adults. However, when the cognitive load increases, it predicts a shift to an overactive or bilateral pattern of activation in young adults to cope with task demands, but under activation in older adults, concomitant with declining performances. This model has been supported by empirical evidence of cognitive aging yielded by studies using functional magnetic resonance imaging (fMRI) [25,26], electroencephalography (EEG) [27], and functional near infra-red spectroscopy (fNIRS) [28,29]. However, there has still been relatively little research on why some older adults show this pattern of compensation, accompanied by preserved or even enhanced cognitive performance, while others do not. According to Reuter-Lorenz and Cappel, “training, exercise, and other interventions {…} may increase available resources and compensatory potential” [23] (p. 180). The effect of one of these interventions, namely physical exercise, and its positive impact on cardiorespiratory fitness (CRF), was assessed in the present study.

Physical exercise is acknowledged to be one of the most powerful strategies for maintaining general health and cognitive and cerebral vitality [30,31,32,33]. Aerobic exercise and CRF level have been shown to be positively related to cognitive performance in older adults, particularly when executive control processes that enlist the PFC are critical for task success [34,35,36,37,38,39]. Although the fundamental mechanisms responsible for these relationships have yet to be clearly established, numerous interventional studies in animals (for a review, see [40,41]), and some in humans, point to a direct effect of exercise and CRF level on brain structure and functions [42,43,44,45]. Functional neuroimaging studies have shown that high-fit older adults tend to show more youth-like patterns of brain activation, coupled with better executive performance, than their low-fit counterparts [42,46,47]. For example, in a recent study, researchers used fNIRS to examine brain activation in both the left and right dorsolateral PFC (DLPFC) during the Stroop interference task (predominantly eliciting the left DLPFC in young adults), in older men as a function of their CRF level [46]. The authors showed mainly bilateral activation of the DLPFC, consistent with HAROLD. More importantly, they observed considerable interindividual variability, with higher-fit older adults outperforming their lower-fit counterparts on inhibition and recruiting the task-dominant left hemisphere more to sustain this enhanced executive performance. The authors concluded that CRF level is related to youth-like, lateralized activity, which is associated with higher cognitive functioning. These findings favor the hypothesis that higher CRF levels allow older people to recruit more resources in the task-specialized neural network, rather than compensating for potential neurocognitive deficits by recruiting additional contralateral brain areas, as predicted by the CRUNCH model. However, this conclusion contradicts the literature that shows older adults who performed poorly on a source memory task recruited lateralized PCF regions similarly to young adults, but inefficiently, whereas high-performing older adults recruited PFC regions bilaterally to sustain their better performance [7]. In the field of exercise psychology, researchers used fNIRS to examine brain activation in both the left and right DLPFC during performance of the random generation task (also involving executive functioning and predominantly enlisting the left DLPFC in young adults), in older women as a function of CRF level [36]. As previous study [46] showed, the authors demonstrated mainly bilateral DLPFC activation and better executive performance among the high-fit participants. However, this better cognitive performance was mediated by increased recruitment of the contralateral (right) DLPFC in these high-fit older women, thus favoring the notion of compensation suggested by the CRUNCH model. 

To ascertain whether CRF level is related to better executive performance because of the preservation of a youth-like pattern of lateralized brain activation or because of compensation in the form of additional contralateral brain recruitment, and to further test the CRUNCH model, cognitive load needs to be manipulated experimentally, with at least three levels of task demand [23]. To the best of our knowledge, this has never been reported in the literature, and the aim of the present study was thus to explore the link between PFC activation and cognitive performance as a function of cognitive load, chronological age, and CRF level. More specifically, we administered an *N*-back task under three conditions of increasing cognitive load and examined brain activity in the bilateral PFC using fNIRS. We chose the *N*-back task because it evaluates working memory updating, a core executive function that has been shown to be impaired in the older population [25,28,48], but better at high CRF levels [49,50]. fNIRS, an optical neuroimaging method that noninvasively monitors the cerebral hemodynamics of oxygenated (O_2_Hb) and deoxygenated (HHb) hemoglobin, has proved its suitability for measuring PFC activation during this type of cognitive task and its sensitivity to the effects of chronological age [28,29] and CRF level [36,51] for a recent review, see [52]. 

Based on the aforementioned studies and the predictions of the CRUNCH model, we expected PFC activation in older adults to increase bilaterally under the two lowest cognitive loads, and to decrease in the most complex condition, concomitantly with a decline in performance. By contrast, young adults would demonstrate more lateralized task-specific activation of the right PFC under the low cognitive loads, and would rely more on increased bilateral PFC activation under the highest cognitive load to sustain their cognitive performance. If CRF level does indeed favor compensation, then the older high-fit adults would exhibit a similar change in activation patterns as a function of cognitive load, and would perform better than the low-fit older adults under the highest cognitive load. 

## 2. Method

### 2.1. Participants 

Participants were 19 young adults aged 18–22 years and 37 older adults aged 60–77 years. They were all right-handed according to the Edinburgh Handedness Inventory [53]. None of them had cardiovascular, neurological or rheumatoid disease. We used the French version of the Mini Mental State Examination (MMSE; [54]) to assess the older adults’ overall cognitive functioning (individual scores ranged between 26 and 30). In addition, we checked the absence of depressive symptoms with the French version of the Geriatric Depression Scale (GDS; [55]). Older adults with an MMSE score <26 and/or a GDS score >10 were excluded from the study, as well as those using medication that could affect cardiorespiratory or cognitive functions (see [56]). All participants provided their written informed consent, and the study was approved by the local ethics committee (no. 2015-04-02) in accord with the ethical standards laid down in the Declaration of Helsinki.

### 2.2. Cognitive Assessment 

We assessed executive performance with the *N*-back task. This task allowed us to assess working memory updating, which is particularly sensitive to age-related decline [57]. We chose it because it had been already used in the literature in both young adults [58,59,60] and older ones [28,48] to examine PFC activation with fNIRS or fMRI. During the *N*-back task, participants are presented with a series of consonants, and have to decide whether each letter is identical to the consonant that preceded it by *N* places in the series [61]. In the present study, we used three cognitive load conditions (1-, 2-, 3-back) and a control condition (0-back) (see [60,61] for a similar procedure). 

### 2.3. CRF Assessment

The young adults were all students from the Sport Sciences Department of the University of Poitiers and were considered to have good-to-excellent CRF (mean VO_2_ max = 54.83 ± 7.21 mL/kg/min) according to a maximal fitness test [62]. The older adults’ level of CRF was determined by the NASA/JSC physical activity scale [63]. Briefly, older adults rated their regular weekly physical practice on a scale of 0–7. The predictive equation developed by these authors allowed us to estimate participants’ CRF level, based on their physical practice ratings and their age, body mass index (BMI) and sex. Previous research had shown that estimated VO_2_ max significantly correlates with measured VO_2_ max (*R* = 0.79; [63]) and accurately distinguishes between high-fit and low-fit individuals [36]. High-fit (VO_2_ max = 26.1 ± 6.7 mL/kg/min) and low-fit (VO_2_ max = 17.4 ± 6.6 mL/kg/min) groups were constituted on the basis of published norms [64]. The characteristics of all participants are summarized in Table 1.

### 2.4. Instrumentation

We recorded relative changes in O_2_Hb and HHb concentrations ({O_2_Hb} and {HHb}) on a two-wavelength (857 and 764 nm) continuous-wave near-infrared spectrometer (OxyMon MkIII; Artinis Medical Systems BV, Zetten, Netherlands). This tool measures relative changes in O_2_Hb and HHb, using the modified Beer-Lambert law [65]. This law takes into account the differential pathlength factor (DPF), which is determined using the following formula: DPF (λ = 807 nm, A) = 4.99 + 0.067 × (age ^0.814^) [66]. In our study, the DPF ranged from 5.69 to 6.60, and data were collected at a sampling rate of 10 Hz. Eight optical channels, comprising four emitters and four receptors, covered the right and left DLPFC and ventrolateral PFC (VLPFC) (Brodmann areas, BAs 9/46 and 47/45/44), which were located using the10/20 international system [67]. The distance between each emitter and receptor was 3.5 cm. During the experiment, participants were asked to avoid making sudden head movements, frowning, clenching their jaw or talking, in order to minimize noise in the hemodynamic signals.

#### fNIRS Data Analysis

**Figure 1:**
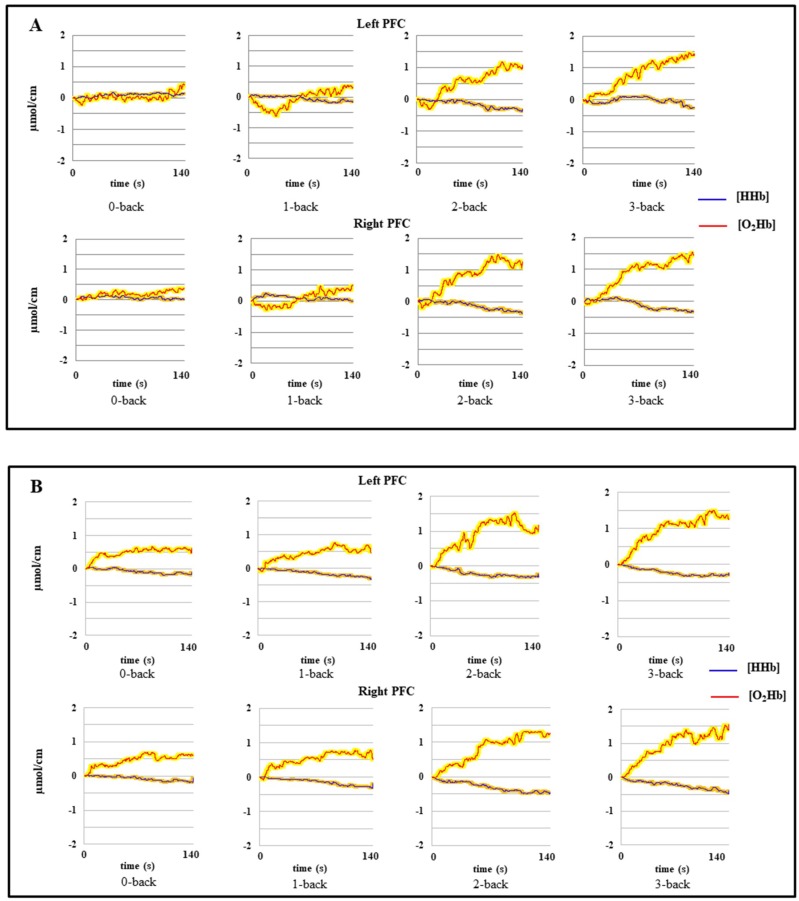

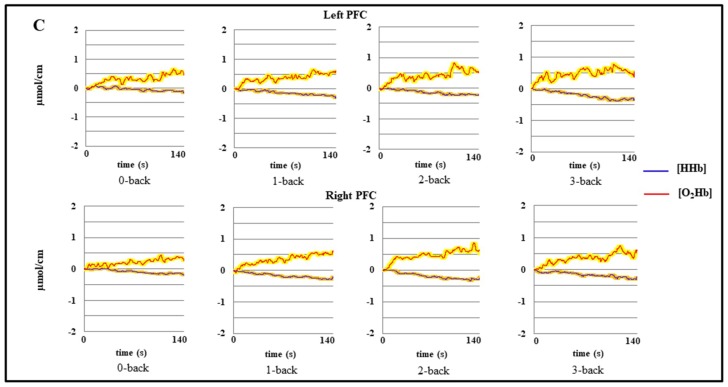
Mean hemodynamic activity as a function of cognitive load, age and hemisphere. (**A**) = Young adults; (**B**) = High-fit older adults; (**C**) = Low-fit older adults. The colored frames on the curves correspond to the standard error of the mean. On the *x* axe, 0 corresponds to the start of the task + 10 s. and 140 correspond to the end of the task (see text).

A moving Gaussian window of 1 s was applied to the raw hemodynamic signals to filter out the noise of the heart beat frequency. We used the movement artifact reduction algorithm (MARA) implemented in MATLAB^TM^ [68] to remove motion artifacts when necessary. After the filtering process, we set the bias to 0 ten seconds after the start of each experimental condition [28]. The mean hemodynamic activity as a function of cognitive load, age and hemisphere is shown in the Figure 1 We then determined the peak response of O_2_Hb and {HHb} during each experimental condition and calculated the mean activation for a 20-s window around each peak (peak ± 10 s; see [69] for a similar procedure). Finally, we subtracted the activation of the control condition (0-back) from that of each experimental condition (see [70]), to measure changes in {O_2_Hb} and {HHb}. Statistical analyses were performed on the differences in mean activation between the control condition and the other experimental conditions (i.e., 1-back–0-back; 2-back–0-back; 3-back–0-back).

### 2.5. Procedure 

Participants were seated in front of a computer screen in a quiet, dimly lit room. Once seated, they performed the *N*-back task in the three cognitive load conditions (1-, 2-, 3-back). The order of the conditions was counterbalanced across participants using a modified Latin square. Each of these three conditions was both preceded and followed by the 0-back condition, the first time as a familiarization and training condition, to ensure good comprehension and eliminate novelty effects, the second time as the control condition. Each condition lasted 150 s. Between conditions, participants were given a 90-s rest period. In each condition, the presentation of the stimuli was controlled by E-Prime^®^ software 2.0 (Psychology Software Tools, Sharpsburg PA, USA), which also recorded response times (RTs) and response accuracy. In each condition, participants were exposed to 42 stimuli (consonants), of which 14 were target stimuli (requiring a “yes” response). Before each stimulus appeared, a black fixation cross was displayed in the center of the screen for 500 ms. In the 1-back, 2-back and 3-back conditions, participants had to decide as accurately and as quickly as possible whether each letter displayed on the screen was identical to the one presented *N* trials before (one, two or three trials before for the 1-back, 2-back and 3-back conditions). In the 0-back condition, participants had to decide whether or not the letter on the screen was an *X*. Participants responded by pressing their left or right index finger on one of two keys (YES/NO) of the millisecond-accurate E-Prime^®^ Serial Response Box™ (Psychology Software Tools, Sharpsburg PA, USA). The interstimulus interval was 3000 ms. We calculated an accuracy score (A´) using the following formula: 0.5 + ((hit rate − false alarm rate) × (1 + hit rate − false alarm rate)) / (4 × hit rate × (1 − false alarm rate)) [71]. At the end of each condition, participants rated the perceived difficulty of the task on a validated 15-point scale [72]. Hemodynamic data were recorded throughout the session. 

### 2.6. Statistical Analysis 

We ran two sets of analyses using STATISTICA software version 12.0 (StatSoft, Paris, France) and R software (Vienna, Austria) [73]. The first comparison was carried out between the young and older adults to determine the effect of chronological age. The second comparison was carried out between the low-fit and high-fit older adults to explore the effect of CRF. Regarding the first comparison, behavioral data (RT, A´, and perceived difficulty) were analyzed with a 2 (group: young vs. older adults) × 4 (cognitive load: 0- vs. 1- vs. 2- vs. 3-back) multivariate analysis of variance (MANOVA), with group as a between-participants factor and cognitive load as a within-participants factor. For the hemodynamic data, the {O_2_Hb} data for the DLPFC and VLPFC were strongly correlated, as were the HHb data (*r* = 0.65–0.76, *p* < 0.05 for right hemisphere; *r* = 0.67–0.74, *p* < 0.05 for left hemisphere) and did not differ significantly. We therefore pooled and averaged the DLPFC and VLPFC data by hemisphere. A 2 (group: young vs. older adults) × 2 (hemisphere: left vs. right) × 3 (cognitive load: 1- vs. 2- vs. 3-back) MANOVA was performed on the averaged {O_2_Hb} and {HHb}. Concerning the second comparison, the same analyses were performed on the behavioral and hemodynamic data, with group (low-fit vs. high-fit older adults) as a between-participants factor. Furthermore, multiple analyses of covariance (MANCOVAs) were performed to control for level of education, which differed significantly between the low-fit and high-fit groups. Whenever necessary, we applied Tukey’s HSD corrections to explore multiple comparisons. Finally, to modeling the relationships between the behavioral data and hemodynamic parameters, we ran linear regression and robust regression analyses on these parameters under the highest cognitive load (3-back). For all these analyses, the level of significance was set at *p* ≤ 0.05, and we report the effect sizes (η^2^ or Wilks′ lambda) for significant effects. 

## 3. Results

### 3.1. Young *vs.* Older Adults

#### 3.1.1. Behavioral Data

The MANOVA revealed a main effect of cognitive load on perceived difficulty, *F*(3, 52) = 230.56, *p* < 0.05, Wilks′ lambda = 0.06, indicating that perceived difficulty increased linearly as a function of cognitive load in both groups (see Table 2). Post hoc analyses indicated a significant difference between 0-back and each of the three cognitive load conditions (all *p*s < 0.05), as well as between 1-back and 2-back (*p* < 0.05) and 2-back and 3-back (*p* < 0.05). For A´, the MANOVA revealed a main effect of cognitive load, *F*(3, 52) = 113.93, *p* < 0.05, Wilks′ lambda = 0.13, and a significant Group × Cognitive load interaction, *F*(3, 52) = 6.12, *p* < 0.001, Wilks′ lambda = 0.73. Post hoc analyses indicated that the young adults were more accurate than the older ones in the 3-back condition (*p* < 0.05). Concerning RTs, the MANOVA revealed a main effect of cognitive load, *F*(3, 52) = 33.68, *p* < 0.001, η^2^ = 0.55, and a significant Group × Cognitive load interaction, *F*(3, 52) = 8.11, *p* < 0.05, Wilks′ lambda = 0.68. Post hoc analyses indicated that the young adults were faster than the older ones in the 1-, 2- and 3-back conditions (all *p*s < 0.05), but not in the 0-back condition (*p* > 0.05). All behavioral results are summarized in Table 2.

#### 3.1.2. fNIRS Data

The hemodynamic data of one young adult (16/896 signals equivalent to ~1.8%) were unusable and thus were not analyzed. Almost all (93.3%) the remaining hemodynamic data showed a classic activation pattern, with an increase in {O_2_Hb} and a slight decrease in {HHb}.

**Figure 2:**
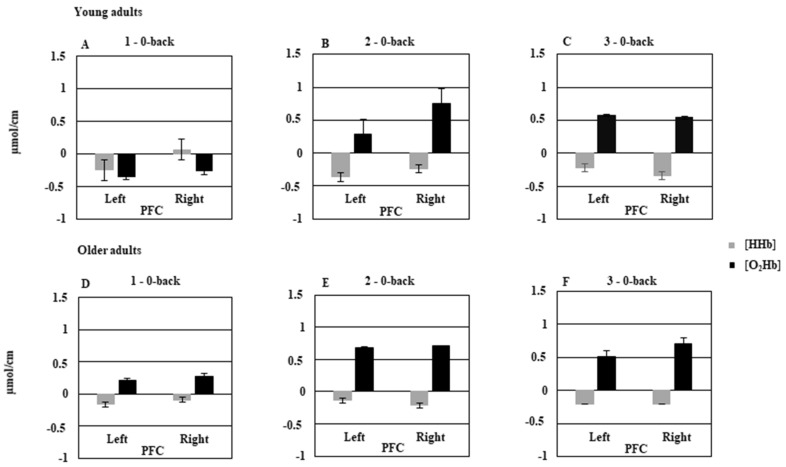
Hemodynamic concentration changes in prefrontal cortex (PFC) of young adults and older adults as a function of cognitive load (**A** = 1–0-back; **B** = 2–0-back; **C** = 3–0-back in young adults and **D** = 1–0-back; **E** = 2–0-back; **F** = 3–0-back in older adults) and hemisphere. Bars represent standard error.

Figure 2 shows mean {O_2_Hb} and {HHb} changes for the 1-back–0-back, 2-back–0-back, and 3-back–0-back contrasts for the young and older adults in the left and right PFCs. The MANOVA performed on relative changes in {O_2_Hb} showed a main effect of cognitive load, *F*(2, 52) = 14.76, *p* < 0.05, Wilks′ lambda = 0.63, and a significant Group × Cognitive load × Hemisphere interaction, *F*(2, 52) = 4.38, *p* < 0.05, Wilks′ lambda = 0.85. This interaction indicated that activation in both hemispheres was minimal in the young group under the 1-back condition (see Figure 2A), but was greater in the right hemisphere than in the left one under the 2-back condition (*p* < 0.05; see Figure 2B), and equivalent for both hemispheres (*p* = 0.99; see Figure 2C) under the 3-back condition. Concerning the older adults, we observed significantly greater activation than for the young adults under the 1-back condition (*p* < 0.05; see Figure 2D). It increased under the 2-back condition, and stabilized in the 3-back condition. The two hemispheres were equally activated across all three cognitive load conditions (all *p*s > 0.05). Finally, when we compared the young and older adults on activation across the hemispheres under the 2-back and 3-back conditions, we found no significant difference between the two groups (both *p*s > 0.05). The MANOVA performed on relative changes in [HHb] showed that the main effect of cognitive load was only close to being statistically significant, *F*(2, 52) = 2.94, *p* = 0.061, Wilks′ lambda = 0.89. However the Group × Cognitive load × Hemisphere interaction was significant, *F*(2, 52) = 5.87, *p* < 0.05, Wilks′ lambda = 0.81. This result indicated that in young adults, {HHb} was significantly greater in the right hemisphere than in the left one (*p* < 0.05) under the 1-back condition. This difference was not significant under the 2-back and 3-back conditions. In the older adults, no significant difference was found between the two hemispheres as a function of cognitive load.

### 3.2. Low-Fit *vs.* High-Fit Older Adults

#### 3.2.1. Behavioral Data

The MANOVAs conducted on perceived difficulty and RTs failed to reveal any significant effect of group, but showed a significant effect of cognitive load on both perceived difficulty, *F*(3, 32) = 13.32, *p* < 0.05, Wilks′ lambda = 0.44, and RTs, *F*(3, 31) = 4.15, *p* = 0.013, Wilks′ lambda = 0.71. The MANOVA conducted on A´ showed a main effect of cognitive load, *F*(3, 33) = 109.52, *p* < 0.05, Wilks′ lambda = 0.09, and a significant Group × Cognitive load interaction, *F*(3, 33) = 2.91, *p* = 0.04, Wilks′ lambda = 0.79. This interaction indicated that high-fit older adults were more accurate than the low-fit ones, but only during the 3-back condition (see Table 2). Entering level of education in the MANCOVA as a covariate reduced the significance of the Group × Cognitive load interaction to a trend level, *F*(3, 32) = 2.4, *p* = 0.08, Wilks′ lambda = 0.81. 

#### 3.2.2. fNIRS Data

**Figure 3:**
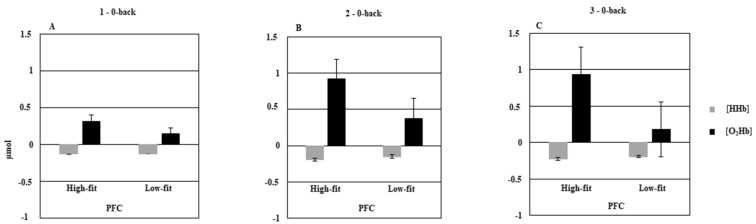
Hemodynamic concentration changes in prefrontal cortex (PFC) in low-fit and high-fit older adults as a function of cognitive load (**A** = 1–0-back; **B** = 2–0-back; **C** = 3–0-back). Bars represent standard error.

The MANOVA conducted on relative changes in [O_2_Hb] showed a significant Group × Cognitive load interaction, *F*(2, 34) = 4.2, *p* = 0.02, Wilks′ lambda = 0.8. This interaction remained significant even after we controlled for level of education with the MANCOVA, *F*(2, 33) = 3.72, *p* = 0.034, Wilks′ lambda = 0.81. This result indicated that the high-fit older adults exhibited significant {O_2_Hb} increases in both hemispheres under the 2-back (*p* < 0.05) and 3-back (*p* < 0.05) conditions, compared with the 1-back condition (see Figure 3A–C). Conversely, the low-fit older adults displayed less activation overall than the high-fit older adults, and no significant differences between the three cognitive load conditions (all *p*s > 0.05). The MANOVA conducted on relative changes in {HHb} showed a significant Group × Hemisphere interaction, *F*(1, 35) = 5.49, *p* = 0.02, Wilks′ lambda = 0.86. This interaction remained significant even after we controlled for level of education with the MANCOVA, *F*(1, 34) = 10.11, *p* = 0.003, Wilks′ lambda = 0.77. This interaction indicated that overall, there was a greater {HHb} decrease only in the left hemisphere of the high-fit older group (−0.14 ± 0.57 µmol/cm) than in that of the low-fit older group (−0.2 ± 0.49 µmol/cm).

### 3.3. Relationships between Behavioral Data and Hemodynamic Parameters during the 3-Back Condition

**Figure 4:**
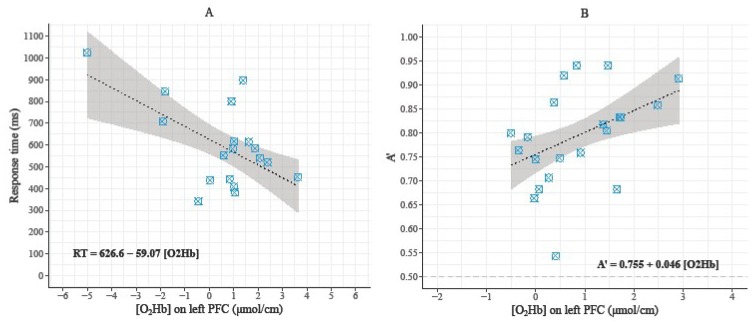
Scatter plots of the robust regressions where {O_2_Hb} in the left PFC predicted behavioral performance in young adults (**A**) and high-fit older adults (**B**) during the 3-back condition. The grey frame represents the 95% confidence intervals.

Even if the analyses did not detect outliers, we performed both normal and robust regression analyses due to extreme points that can influence the models [74]. In the 3-back condition, the dependent variable was the behavioral performance (RT for the young adults and A’ score for the high-fit older adults) and the predictor was {O_2_Hb} changes in the left PFC (see Figure 4A,B). For young adults, RT was significantly predicted by {O_2_Hb} changes in the left PFC (β for the normal regression = −54.53; β for the robust regression = −59.0697; *R*² = 0.3065; *r* = −0.55; *p* = 0.017). For the high-fit older adults, A’ score was significantly predicted by {O_2_Hb} changes in the left PFC (β for the normal regression = 0.04683; β for the robust regression = 0.0458; *R*² = 0.1868; *r* = 0.43; *p* = 0.05). Concerning the low-fit older adults, no significant relationship was found between behavioral data and {O_2_Hb} changes in the PFC. Finally, no significant relationship was found between {HHb} changes and behavioral data.

## 4. Discussion

The present study was intended to examine the effects of chronological age and CRF on cognitive performances and prefrontal activation patterns as a function of cognitive load, in order to further test the influence of CRF level on the predictions of the CRUNCH model [23]. The main findings were that chronological age has adverse effects on cognitive performance and prefrontal cortex activation as cognitive load increases. For higher levels of cognitive load, however, CRF compensates for these behavioral and brain activation declines. 

### 4.1. Effect of Age on Behavioral Data and Prefrontal Hemodynamic Activity

Perceived difficulty of the cognitive tasks significantly increased as a function of cognitive load and was equivalent across young and older adults. This result indicates that age did not affect perceived difficulty, which was probably not therefore responsible for the differences observed in behavioral performance and prefrontal activation. Results also showed that young adults responded faster than older adults under all three cognitive load conditions. Moreover, they were significantly more accurate than the older adults under the greatest cognitive load (3-back) condition. Although Vermeij and colleagues only manipulated two levels of cognitive load (1-back and 2-back), our results converge with their finding that older adults are slower than young adults, but only demonstrate a significant decline in accuracy in the most complex condition of a very similar experimental task [28]. Overall, our results are in agreement with the abundant literature showing age-related declines in processing speed and working memory updating [75,76,77,78]. At the behavioral level, these results support the prediction of the CRUNCH model that increasing cognitive load will induce an age-related deterioration in executive performance [23,25,48]. 

The measurements of hemodynamic activity in the young adults indicated minimal PFC activation under the 1-back condition, increased right-lateralized activation under the 2-back condition, and bilateral activation under the 3-back condition (highest cognitive load). As such, these results are in line with the predictions of the CRUNCH model. However, in older adults, hemodynamic activity only partially supported the predictions of the CRUNCH model. Although we observed clear bilateral overactivation under the lower levels of cognitive load, consistent with predictions, we did not observe the predicted strong reduction in PFC hemodynamic activity in the older adults under the 3-back condition. This result suggests that the older adults in our study were able to maintain quite a high level of PFC activation that enabled them to compensate for the deactivation that theoretically occurs. However, this compensation did not allow them to maintain their behavioral performance. There are two possible explanations for this result. First, the older group had the same subjective perception of task difficulty as the young group, and this perceived difficulty was not given the maximum rating under the 3-back condition (11.5/15 for the older adults and 10.7/15 for the young ones). We can therefore argue that the cognitive load induced by the 3-back condition may not have been sufficient to induce this deactivation. Second, many of the older adults included in our study regularly practiced physical exercise and had a good-to-excellent CRF level, which is one of the factors that can improve compensatory potential [23,36,51]. Accordingly, the considerable variability we observed in the data for this population suggests that the patterns of behavioral and hemodynamic data may differ according to CRF level. 

### 4.2. Effect of CRF Level on Behavioral Data and Prefrontal Hemodynamic Activity

The comparison between the low-fit and high-fit older adults showed that at the behavioral level, perceived difficulty and RT did not differ between the two groups. By contrast, the high-fit older adults were more accurate than their low-fit counterparts under the 3-back (highest cognitive load) condition, even if level of education partly explained this superiority. The effect of CRF level on executive performance was consistent with the findings of previous studies involving other working memory tasks, which showed that high-fit older adults have better working memory updating performance than low-fit older adults [34,49]. Regarding hemodynamic activity, we found that the high-fit older adults exhibited a significant and bilateral increase in PFC activation as a function of cognitive load, whereas the corresponding response in the low-fit older adults was compromised. More specifically, the high-fit older adults showed an increase in PFC activation across the 1-back and 2-back conditions that stabilized under the 3-back condition (no significant increase or decrease). The latter pattern of activation was closer to that of the young adults in the present study than to that of the low-fit older adults, who showed less overall PFC activation and no significant variations as a function of cognitive load. Although they do not completely validate the strong predictions of the CRUNCH model, these results do lend some support to the compensation hypothesis whereby overactivation or bilateral recruitment of brain regions is functionally related to better cognitive performances. They are in line with previous results showing that higher levels of CRF are related to increased PFC activation, which mediates better executive performance [36,51]. In other contexts, neuroimaging studies have reported similar results, with high-performing older adults over recruiting bilateral brain regions to maintain their cognitive superiority over low-performing older adults in various cognitive domains such as episodic memory and working memory [7,27,79]. Although the reasons why some participants were high performers and others low performers were not explained in these studies, we can hypothesize that these older adults diverged on CRF level, underlining the importance of examining this factor in cognitive neuroimaging studies. The substantial PFC hemodynamic activity observed in the high-fit older adults could be explained by the cardiorespiratory hypothesis whereby an increased level of CRF increases regional cerebral blood flow (rCBF), thus ensuring a better oxygen supply to the brain, particularly in the prefrontal regions where there is the greatest age-related deficit in rCBF, and translates as improved executive performance [80,81,82].

### 4.3. Relationships between Behavioral Data and Prefrontal Hemodynamic Activity 

One important result of the present study was the significant relationship between behavioral data and {O_2_Hb} changes under the 3-back condition in the young and high-fit older groups, but not in the low-fit older group. {O_2_Hb} changes in the left PFC predicted RT in the young adults and predicted A´ in the high-fit older adults. Although the proportion of variance explained by changes in {O_2_Hb} was not very important, these results suggest that the better performances observed among these participants were functionally related to the hemodynamic activity of their left PFC, which is the hemisphere where there was additional activation under this high cognitive load condition. This positive relationship between behavioral performance and {O_2_Hb} changes in the high-fit older adults’ PFC agrees with and extends the results of previous study, which showed that the better executive performances of older women are mediated by the over recruitment of the right (contralateral) DLPFC, using a random generation task that predominantly recruited the left DLPFC [36]. Taken together, these results also lend some support to the compensation hypothesis.

### 4.4. Limitations

The present study has several limitations that need to be outlined. First, its cross-sectional design did not allow us to formally establish a direct causal link between CRF level, hemodynamic activity and executive performance. Although this cross-sectional design was needed to ensure that we had found tasks that were suitable and sufficiently sensitive for our measurement procedure, randomized controlled trials will be needed in the future to establish this causal link. Another limitation is that the material and mapping used in the present study did not allow us to explore the hemodynamic activity of the entire brain. Indeed, the principal limitations of continuous wave NIRS concern its relatively small spatial resolution, comparatively to another metabolic brain imaging techniques, and its incapacity to measure the deeper sub-cortical hemodynamic activity [52,83]. Thus, the results obtained in the present study only concerned the PFC, which was strongly elicited by our experimental task. In addition, we did not control for either superficial skin blood flow or systemic effects in our fNIRS signals. A recent opinion article underlined the importance of controlling for these extra cortical contributions to fNIRS signals to optimize the interpretation of the hemodynamic response [84]. However, as activation patterns were obtained in the present study by subtracting the activation of the control condition from that of each experimental condition, and because the different cognitive loads were counterbalanced, any overall systemic response should have been neutralized. Third and last, a second control group composed of young adults with a low level of CRF would have enabled us to better explore the combined effects of age and CRF level on cognitive performance and brain activation. 

## 5. Conclusions

In conclusion, the results of the present study were in line with the behavioral predictions of the CRUNCH model, and showed a significant deterioration in executive performance among older adults compared with young ones, as a function of task demands. Regarding brain activity, our results partially confirmed the predictions of the CRUNCH model and suggested that CRF modulates the brain activation patterns of older adults during an executive task. Further interventional studies are needed to confirm our findings and determine the characteristics of a training program to counteract the reduction in brain activity that is assumed to be at the core of the age-related cognitive decline. Based on the review literature, different training programs involving combination of aerobic and strength exercises [37] or specific motor activities improving sensorimotor fitness [44] should demonstrate qualitatively different cognitive and cerebral gains, allowing to propose specific hypotheses on their influence on brain efficiency and connectivity. Finally, to more deeply evaluate the compensation hypothesis and the functional role of over or bilateral cerebral activations, future studies should examine the hemodynamic activity of the entire cerebral cortex using modern approaches of functional brain connectivity [85,86]. 

## Figures and Tables

**Table 1:** Characteristics of the participants.

	Young Adults (*N* = 19)	Older Adults (*N* = 37)	Older High-Fit (*N* = 21)	Older Low-Fit (*N* = 16)	*p* Value
Age (years)	19.7 ± 1	68.95 ± 4.74	67.90 ± 4.86	70.31 ± 4.33	*p* = 0.12
Gender (M/F)	17/2	15/22	8/13	7/9	*p* = 0.79
Education (years)	14.00 ± 0.00	13.35 ± 3.85	14.52 ± 3.57	11.81 ± 3.75	*p* = 0.031 *
VO_2_max (mL/Kg/min)	54.83 ± 7.21	22.31 ± 7.88	26.10 ± 6.73	17.40 ± 6.58	*p* = 0.0004 *
MMSE		29.24 ± 0.95	29.29 ± 1.01	29.19 ± 0.91	*p* = 0.76
GDS		5.78 ± 4.25	5.95 ± 4.86	5.56 ± 3.42	*p* = 0.78

* Significant difference between high-fit and low-fit older adults; MMSE = Mini Mental State Examination; GDS = Geriatric Depression Scale.

**Table 2:** Evolution of behavioral data as a function of cognitive load and group.

		Young Adults (*N* = 19)	Older Adults (*N* = 37)	Older High-Fit (*N* = 21)	Older Low-Fit (*N* = 16)
RT (ms)	0-back	366.77 ± 43.46	509.41 ± 72.79	499.84 ± 67.38	521.00 ± 79.95
	1-back	404.39 ± 73.68	661.39 ± 161.77 *	655.22 ± 172.32	669.50 ± 151.95
	2-back	517.46 ± 152.98	970.15 ± 316.46 *	959.12 ± 304.53	984.63 ± 341.04
	3-back	592.72 ± 186.32	1110.80 ± 370.47 *	1114.56 ± 412.97	1105.86 ± 319.30
A’	0-back	0.99 ± 0.01	0.99 ± 0.01	0.99 ± 0.01	0.99 ± 0.01
	1-back	0.98 ± 0.02	0.96 ± 0.04	0.97 ± 0.03	0.95 ± 0.05
	2-back	0.93 ± 0.06	0.90 ± 0.09	0.90 ± 0.07	0.89 ± 0.12
	3-back	0.85 ± 0.06	0.75 ± 0.16 *	0.79 ± 0.1	0.69 ± 0.20 **
Perceived difficulty	0-back	2.24 ± 1.17	2.51 ± 1.26	2.33 ± 1.35	2.75 ± 1.13
	1-back	4.42 ± 2.17	5.24 ± 1.94	5.24 ± 2.21	5.25 ± 1.57
	2-back	7.74 ± 1.63	8.51 ± 2.27	8.95 ± 2.16	7.94 ± 2.53
	3-back	10.74 ± 1.99	11.49 ± 1.79	11.86 ± 1.62	11.00 ± 1.93

* Significant difference between young and older adults; ** Significant difference between high-fit and low-fit older adults.
